# OntoTiger: a platform of ontology-based application tools for integrative biomedical exploration

**DOI:** 10.1093/nar/gkaf337

**Published:** 2025-04-29

**Authors:** Haixiu Yang, Guoyou He, Meiyi Zhang, Hongyu Fu, Guanzhi He, Chao Wang, Yangyang Liu, Sainan Zhang, Tao Wang, Yongqun Oliver He, Liang Cheng

**Affiliations:** College of Bioinformatics Science and Technology, Harbin Medical University, Harbin 150081, Heilongjiang, China; College of Bioinformatics Science and Technology, Harbin Medical University, Harbin 150081, Heilongjiang, China; College of Bioinformatics Science and Technology, Harbin Medical University, Harbin 150081, Heilongjiang, China; College of Bioinformatics Science and Technology, Harbin Medical University, Harbin 150081, Heilongjiang, China; College of Bioinformatics Science and Technology, Harbin Medical University, Harbin 150081, Heilongjiang, China; College of Bioinformatics Science and Technology, Harbin Medical University, Harbin 150081, Heilongjiang, China; College of Bioinformatics Science and Technology, Harbin Medical University, Harbin 150081, Heilongjiang, China; College of Bioinformatics Science and Technology, Harbin Medical University, Harbin 150081, Heilongjiang, China; School of Computer Science, Northwestern Polytechnical University, 1 Dongxiang Rd, Xi’an 710072, China; Unit for Laboratory Animal Medicine, University of Michigan Medical School, Ann Arbor, MI 48109, United States; College of Bioinformatics Science and Technology, Harbin Medical University, Harbin 150081, Heilongjiang, China; National Health Commission (NHC) Key Laboratory of Molecular Probes and Targeted Diagnosis and Therapy, Harbin Medical University, Harbin 150028, Heilongjiang, China

## Abstract

Biomedical ontologies, such as Gene Ontology (GO), Disease Ontology (DO), and the Human Phenotype Ontology (HPO), have been extensively applied to characterize molecular roles and their semantic relationships in biomedical research and clinical practice. Although numerous algorithms have been developed to quantify relationships between ontology terms or to explore molecular functions, the absence of a comprehensive tool to integrate these algorithms has limited effective ontology applications. To address this, we developed OntoTiger, a platform of Ontology-based application Tools for InteGrativE biomedical exploRation. OntoTiger combines >20 classic algorithms, supporting six prevalent molecular types as well as five widespread biomedical ontologies. The platform comprises four modules: (i) Annotation module, which qualifies the relationships between ontology terms and molecules; (ii) Similarity module, quantifying functional similarity between/across pairwise ontology terms or between molecules; (iii) Prediction module, characterizing the molecular roles from an ontological perspective; and (iv) Enrichment module, elucidating the potential biological significance of a particular list of molecules. OntoTiger provides a freely accessible, user-friendly web server dedicated to enabling one-stop ontology-based applications and is freely available at https://bio-computing.hrbmu.edu.cn/OntoTiger.

## Graphical Abstract

**Graphical Abstract:**
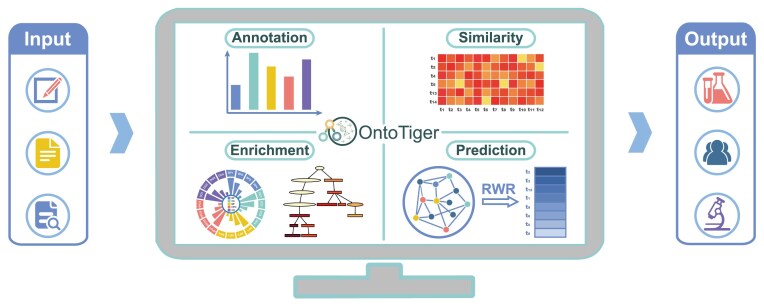

## Introduction

Since Gene Ontology (GO) was established in 2000 [1, 2], hundreds of biomedical ontologies, such as Disease Ontology (DO) [3, 4] and the Human Phenotype Ontology (HPO) [5, 6], have been documented in the BioPortal [7, 8] and the Open Biological and Biomedical Ontology (OBO) Foundry [9, 10]. Benefiting from the human-readable and machine-computable characteristics, many biomedical ontologies have been widely studied and applied in biomedical research.

The annotations associating the molecules with ontology terms describe the function of a molecule from an ontology perspective. Based on these annotations, many computational analyses have been proposed to quantify the relationships between ontology terms and to characterize the roles of molecules. For example, semantic similarity measures were initially applied to quantify ontology terms using annotation information [11–16]. Then, functional associations between molecules were incorporated into the similarity calculation [17]. Furthermore, cosine similarity of semantic vectors was used to improve the performance of the ontology similarity calculation [18–20]. In addition to quantifying the functional relationships between ontology terms, similarity assessment also plays an important role in molecular function prediction [21, 22]. Furthermore, for a particular list of molecules, such as differentially expressed genes (DEGs) or mutated genes, ontology-based enrichment analysis such as over-representation analysis (ORA) [23] and gene set enrichment analysis (GSEA) [24] were used to determine the potential biological significance of this list. To overcome the overenriched problem caused by the “true-path” rule in classic enrichment analysis, TopGO [25] takes the topology of the GO-directed acyclic graph (DAG) into consideration, effectively resolving this problem. While computational efforts greatly accelerate biomedical ontology research, the algorithms remain dispersed and uncoordinated. The absence of a systematic computational platform severely limits the application of biomedical ontologies. Moreover, most algorithms and tools require bioinformatics expertise and programming skills, which may be an obstacle for scientific researchers.

Thus, we developed OntoTiger, a platform of Ontology-based Tools for InteGrativE biomedical exploRation. The platform integrates >20 classic algorithms and develops them into universal tools, accommodating multiple ontologies and molecular types. OntoTiger provides multiple modules that cover the main applications of biomedical ontology, comprising: annotation of ontology–molecule relationships, similarity calculation of pairwise ontology terms/molecules, functional prediction for potential ontology–molecule associations, enrichment analysis for a particular list of molecules, and ID conversion for pre-processing raw data for downstream analysis. OntoTiger (https://bio-computing.hrbmu.edu.cn/OntoTiger) is freely accessible and user-friendly, and supports simple inputs but diverse outputs.

## Materials and methods

### Data collection

OntoTiger currently covers five biomedical ontologies: Biological Process (BP), Molecular Function (MF), Cellular Component (CC), DO, and HPO, as well as six popular molecular types: protein-coding gene (pc_gene), long non-coding RNA (lncRNA) gene, microRNA (miRNA) gene, metabolite, microbe, and drug. The ontology information, including ontology term ID, name, synonyms, and relationships between ontology terms, was collected from GO, DO, and HPO. The concise descriptions of molecular functions and detailed molecular information for genes, metabolites, microbes, and drugs were collected from the Gene Reference Into Function (GeneRIF) (version date: 14/7/2024) database (ftp://ftp.ncbi.nih.gov/gene/GeneRIF/), the human metabolome database (HMDB) [26], gutMDisorder [27], and DrugBank [28], respectively.

### Annotation of molecules with ontology terms

Annotations from ontology resources, such as annotations of pc_gene with the GO term (MF, BP, and CC) and annotations of pc_gene with the HPO term, were collected to describe the function of molecules. In cases where annotations are missing for a specific molecular type and ontology (e.g. annotations of the six types of molecule with DO or annotations of lncRNA and miRNA with HPO), we manually annotate the molecular descriptions with ontology terms.

We firstly extracted all standard DO terms and HPO terms, along with their synonyms, to construct the dictionary. Subsequently, we collected textual descriptions of molecular functions (see “Data collection”). Finally, we used the University of Michigan's Mgrep tool [29], an efficient tool for mapping free text to ontology terms within the NCBO Annotator service [7], to recognize the related ontology concepts in the textual descriptions of molecular functions and to map them to the ontology terms in the dictionary. All annotations linking different molecules to multiple ontology terms were stored in the OntoTiger MySQL relational database management system.

### Similarity calculation of pairwise ontology terms/molecules

OntoTiger adopts >10 algorithms to quantify the relationships between pairwise ontology terms or molecules for the following three submodules. (i) Similarity calculation for pairwise terms of the same ontology. OntoTiger employs nine methods in this module. Among them, Resnik [11], Lin [12], Jiang [13], and Rel [15] are information content (IC)-based methods. The IC of an ontology term is defined as the negative logarithm of its probability of occurrence in the ontology corpus. A rarely used term contains a greater amount of information, and the similarity between two terms depends on their frequencies and the closest common ancestor term in a specific corpus of ontology annotations. Wang [30] is a graph-based method which computes the semantic similarity based on both the locations of these terms in the DAGs of the ontology and their relationships with their ancestor terms. SemFunSim [17] is both a semantic and a function-based method that integrates the relationship between two ontology terms (semantic similarity) and the function of ontology-annotated gene sets in the weighted human gene network (functional similarity). OWL2Vec [31], Obo2Vec [19], and Onto2Vec [20] are machine learning-based methods. They compute the cosine similarity of semantic vectors of pairwise terms and these vectors are constructed with a word-embedding tool that uses feature sequence information and ontology annotation conjointly. (ii) Similarity calculation for pairwise terms across ontologies. OntoTiger applies VSM [32] and CroGO [33] methods in this module. VSM uses the vector space model based on the co-occurrence of ontology terms in annotation databases and association rule mining, while CroGO incorporates genome-specific gene co-function network information. (iii) Similarity calculation for pairwise molecules. In this module, OntoTiger uses PBPA [34] and PAPM [35] methods, combined with the nine methods used in (i). The molecule is represented by its annotated ontology term set, and the similarity of pairwise molecules is calculated by the corresponding term sets. Firstly, one of the nine methods is used to calculate the similarity between each pairwise ontology term from two term sets, then the PBPA or PAPM integrates these similarity scores into a single similarity score.

### Prediction of new molecule–ontology relationships

OntoTiger predicts new molecular functions by fully exploiting the similarities between ontology terms [21]. Firstly, an ontology similarity network is constructed using the similarity calculation method mentioned above, where nodes indicate ontology terms and weighted edges mean the similarity between two ontology terms. Then the random walk with restart (RWR) method [36] is applied on the ontology similarity network, with the known ontology terms annotated to the molecule serving as seed nodes. Candidate molecule–ontology relationships are prioritized, and the top-ranked ontology terms are considered potential functions of the molecule. Similarly, ontology-related molecules can be prioritized using a molecular similarity network.

### Enrichment analysis for the molecular list

OntoTiger provides three ontology enrichment analysis methods, namely classic, weighted, and GSEA. The classic enrichment analysis is an ORA that measures the statistical proportion of an interesting molecular list and specific molecular sets (ontology term). The weighted method evaluates the significance of each term by computing *P*-values from a weighted contingency table (Supplementary Table S1). It addresses the overenriched problem in enrichment analysis by integrating ontology graph topology on a global scale and down-weighting molecules in less significant terms [25]. These two methods incorporate five statistical test models (hypergeometric test, Fisher's exact test, binomial test, log-odds ratio, and χ^2^ test) and seven hypothesis tests [Bonferroni, Holm, Hochberg, Hommel, Benjamini–Hochberg (BH), false discovery rate (FDR), and Benjamini & Yekutieli (BY)]. The GSEA method is a knowledge-based enrichment analysis method, which integrates gene expression data by applying the Kolmogorov–Smirnov test. GSEA aims to determine whether the annotated molecules of ontology terms are randomly distributed throughout the ordered list of DEGs or primarily found at the top or bottom.

### Implementation

OntoTiger is developed as a Frontend–Backend Separation Project using Vue (v3.2.45) for the front end, Spring Boot (v2.5.1) for the back end, and MySQL (v5.7.24) as the database. Vue handles page visualization and interaction with backend data. Spring Boot manages business logic and data processing. All data and the relationships were stored in the MySQL relational database management system, and the functional tools were implemented through R scripts. HTML5, CSS, Axios, jQuery, TypeScript, and Element-Plus were used for web rendering and interaction. Echarts and Vxe-table were used for display and visualization of the results. The OntoTiger website is freely accessible online without registration, providing an easy-to-use web server for comprehensive applications of biomedical ontologies.

Detailed descriptions of the algorithms used in modules of similarity, prediction, and enrichment are provided in the Supplementary data.

## Results

### Overview of OntoTiger and interplay between modules

**Figure 1.**
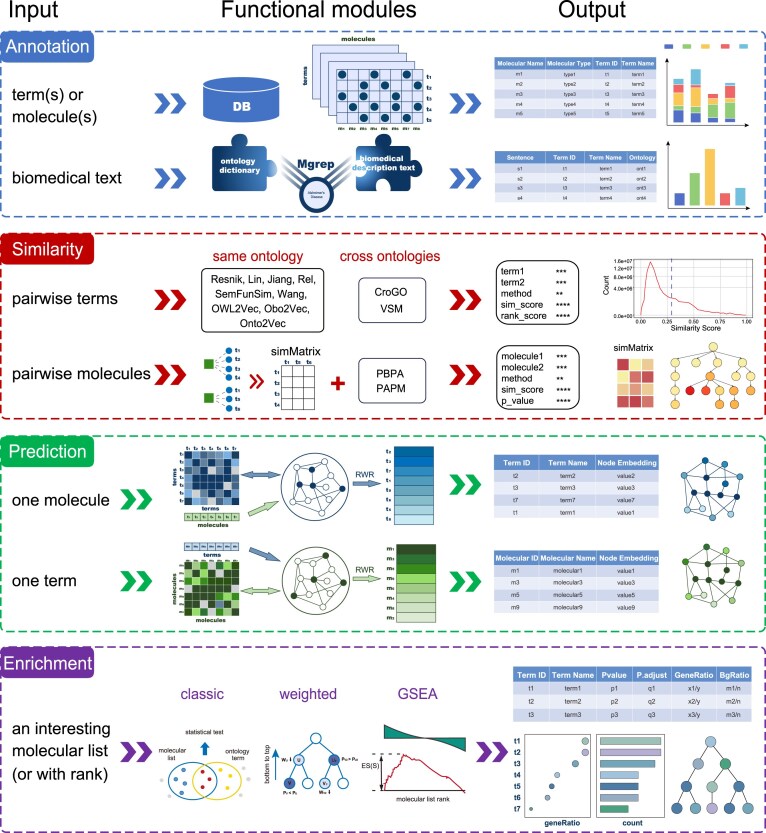
Overview of OntoTiger.

OntoTiger presents a comprehensive platform for one-stop application of biomedical ontology. It mainly comprises five functional modules: Annotation, Similarity calculation, Functional prediction, Enrichment analysis, and ID converter. The Annotation module enables qualitative assessment of molecule–ontology associations through query or annotation functions. The Similarity calculation module provides quantifications of the relationships of pairwise ontology terms or pairwise molecules. The Functional prediction module enables users to identify new molecule–ontology relationships. The Enrichment module provides statistical models to characterize the potential biological significance for a particular molecular list from an ontological perspective. In addition, the input of molecule(s) or ontology term(s) can be standardized by the ID converter module for downstream analysis. Among them, the Annotation and ID converter modules focus on foundational data tasks. They are mainly responsible for data querying, data pre-processing, and the preparation of data for subsequent analysis. The other three modules are functional modules for ontology analysis and application. Each module offers multiple classical algorithms for comprehensive ontology analysis, supporting various molecular types and biomedical ontologies. To simplify the application of each module, we have specified methods with default parameters that are suitable for most analyses. We also encourage users to tune the parameters according to their input data for individual and optimal results. Most importantly, OntoTiger features a user-friendly interface with straightforward input and diverse data output options, which facilitates the complex analysis and function research of ontology. The overview of OntoTiger is shown in Fig. 1.

It is worth noting that the modules are progressive and inter-related. The Annotation module records the ontology–molecule annotations, which supplies data foundational for all the functional modules. Based on annotation information, the Similarity calculation module supplies >10 methods to quantify the similarity of pairwise ontology terms/molecules. Furthermore, all similarities of pairwise terms (or molecules) together form a similarity network, which is used for the Functional prediction module along with the annotation information. Finally, the ontology–molecule annotations are semantically expanded in the ontology DAG according to the hierarchical structure. Based on this DAG, the Enrichment analysis module applies three types of methods to elucidate the underlying biological significance of a molecular list.

### Foundational databases of OntoTiger

**Figure 2.**
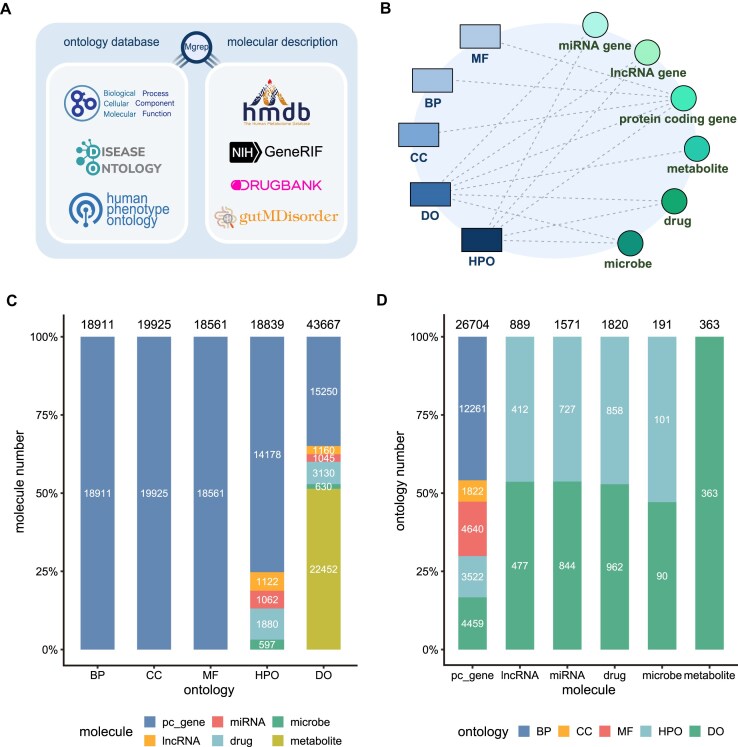
Ontology–molecule annotations of OntoTiger. (**A**) The sources of ontologies and molecular functional descriptions; (**B**) the relationships between molecules and ontologies; (**C**) statistics of the proportion of different types of molecules annotated by each ontology; and (**D**) statistics of the proportion of different ontology types annotated to each molecular type.

OntoTiger maintains a comprehensive database organized into three different themes, i.e. annotations of molecules with ontology terms, similarity for each pair of ontology terms (or molecules), and the structured DAG of ontology with semantic expansion. For the first scheme, the ontology–molecule annotations of OntoTiger encompass six molecular types, as well as five widely used biomedical ontologies (Fig. 2A, B). The annotation database stores a large number of annotations in a MySQL relational database, shown in Fig. 2C and D (Supplementary Table S2).

The following theme is the similarity network. In each ontology network, terms represent nodes, and edges are defined by pairwise similarity scores between terms. For molecules, the pc_gene network and metabolite network are derived from the HumanNet v3 [37] and STITCH 2 [38] databases, respectively. Similarity networks for lncRNA, miRNA, drug, and microbe are constructed by calculating the pairwise similarity scores between molecules. All ontology and molecular similarity networks are stored as RData files in OntoTiger and are utilized in the Prediction module.

The last theme is the ontology DAG with semantic expansion of annotations. Biomedical ontology always has a hierarchical structure that forms a DAG, which follows the “true-path” rule. Thus, we semantically expand the annotations in ontology DAG, where nodes represent ontology terms and edges represent “is_a” relationships (or “part_of” relationships). Nodes with no annotated molecules are pruned. Additionally, to adapt to the weighted method, each node in the DAG is assigned a level according to the length of the longest path from the root. The structured DAGs with semantic expansion of annotations are stored as RData files in OntoTiger and are used in the Enrichment analysis module.

### The Annotation module qualifies the molecule–ontology relationships

The Annotation module allows users to query the ontology–molecule annotations by inputting simple molecule(s) or ontology term(s), and the qualified ontology–molecule relationships would return as a downloadable table and an intuitive visible bar plot. In addition, the “online annotation” function module allows users to annotate a biomedical text with specified ontologies with just clicks of the mouse.

As a demonstration, we queried annotations for DO terms “Alzheimer's disease” (AD), “tauopathy”, and “mild cognitive impairment” (MCI). AD is a brain disease characterized by memory lapses, confusion, emotional instability, and progressive loss of mental ability. Tauopathy refers to a neurodegenerative disease with pathological aggregation of the tau protein in neurofibrillary tangles (NFTs) in the human brain. MCI is a cognitive disorder characterized by objective impairment in cognition with minimal impairment of their capacity to undertake the instrumental activities of daily living. When we queried the annotations for AD with the “Query annotations” module by inputting a simple DO term “Alzheimer's disease”, the results included a list of various types of molecules annotated with AD in the form of a downloadable table and the visualized statistics of results in a bar plot. AD was annotated with 1669 molecules, including 1388 pc_genes, 78 miRNAs, 18 lncRNAs, 127 metabolites, 49 drugs, and 9 microbes (Supplementary Table S3). The finding is consistent with the fact that pc_genes comprise a large number of widely studied genes. Query annotations for tauopathy and MCI were obtained in the same way (Supplementary Table S3). It is worth noting that “tauopathy” has no more annotated genes than AD, even though AD has an “is_a” relationship with “tauopathy”. This discrepancy arises because the results return only those molecules that are directly annotated with the ontology term.

### The Similarity module quantifies the relations of pairwise ontology terms/molecules

**Figure 3.**
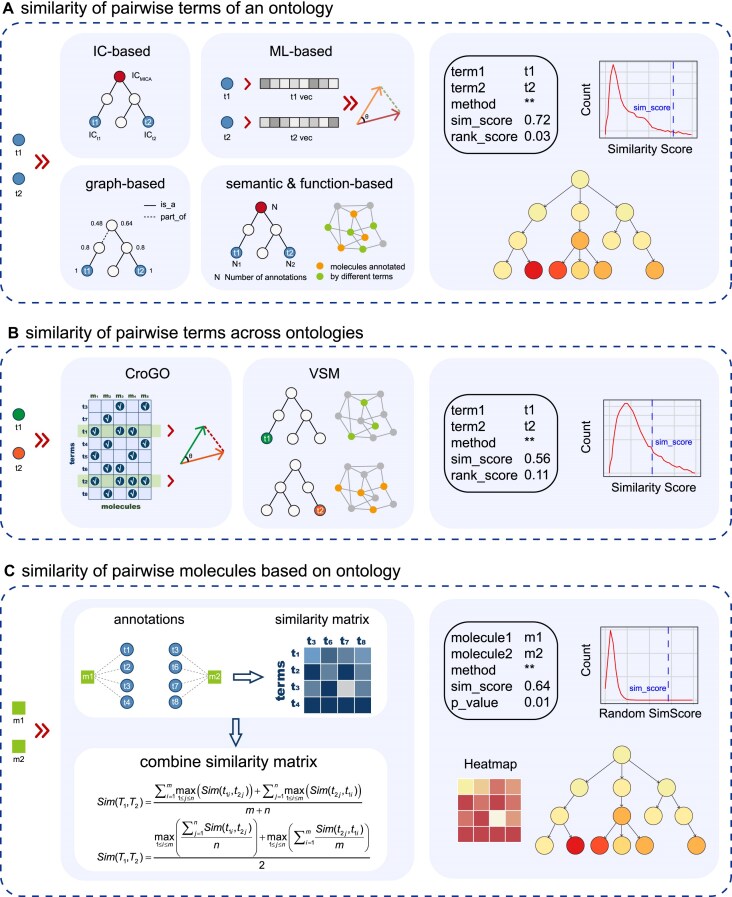
OntoTiger similarity module. Schematic diagrams illustrating (**A**) the submodule for “similarity of pairwise terms of an ontology”; (**B**) the submodule for “similarity of pairwise terms across ontologies”; and (**C**) the submodule for “similarity of pairwise molecules based on ontology”.

The Similarity calculation module facilitate users in quantifying the functional similarity of pairwise ontology terms or pairwise molecules. OntoTiger provides three submodules to perform similarity calculation (shown in Fig. 3). (i) Similarity of pairwise terms of an ontology (Fig. 3A). This submodule enables quantification of the similarity for two terms of an ontology, such as two disease terms or two phenotype terms. (ii) Similarity of pairwise terms across ontologies (Fig. 3B). This submodule provides functions to quantify the relationships of pairwise terms across ontologies, e.g. a disease and a biological process, significantly increasing interoperability between molecules in multiple aspects. (iii) Similarity of pairwise molecules based on ontology terms (Fig. 3C). This submodule enables calculation of the functional similarity between pairwise molecules by aggregating similarities of ontology term sets. The similarity module supports simple input in terms of two ontology terms (or two molecules), and returns similarity scores along with intuitive diagrams.

Since we have obtained annotations of AD, tauopathy, and MCI previously, based on these annotations or DAG topology, the “similarity of pairwise terms of an ontology” submodule enables quantification of the degree of similarity between AD and tauopathy, as well as AD and MCI. By inputting two ontology terms, “Alzheimer's disease” and “tauopathy” (or “mild cognitive impairment”), OntoTiger provides nine different methods to calculate the similarity between two diseases. The results include two scores and two graphical presentations. The similarity score quantifies the relationships between AD and tauopathy (or MCI) terms, which are displayed in Supplementary Table S4. Among these nine methods, the first six methods are based on IC or leverage of the topological structure of DAG. As a result, the similarity score of AD–tauopathy pairs is significantly higher than that of AD–MCI pairs. The subgraph of DAG intuitively illustrates the topological relationship between these two terms in DO DAG. In contrast, the latter three methods utilize term semantic vectors: the OWL2Vec and Obo2Vec methods exhibit comparable scores, while the Ont2Vec method yields a non-applicable result due to the lack of records of MCI.

Quantification of the relationships between AD and other ontology terms, such as biological process, or phenotype, can be done by the “similarity of pairwise terms across ontologies” submodule.

### The Prediction module prioritizes the candidate molecule–ontology relationships

The functional prediction module enables users to characterize the molecular roles from an ontology perspective. On the one hand, users can identify new molecule-related ontology terms by merely inputting a single molecule. OntoTiger then returns a downloadable table and a visual subnetwork of the candidate ontology terms ranking with the probability scores. On the other hand, users can also identify new ontology-related molecules by inputting an ontology term in the same way. The identified new molecule–ontology term relationships indicate the new functions of the molecule.

For example, the known annotations of AD can be used to predict new relationships between AD and other molecules, such as AD-related pc_genes via the “Prediction” module. When we entered the DO term “Alzheimer's disease” and chose the “pc_gene” as molecular type, 1388 known annotated pc_genes of AD were used as seed nodes, and the RWR was then applied to the pc_gene network. The results returned a list of candidate genes ranked by the RWR scores, and the top ranked genes were deemed as potential AD-related genes. Among the top 10 candidate genes (*HRAS*, *HNRNPL*, *ELAVL1*, *GRB2*, *PIK3R1*, *VIRMA*, *KRAS*, *NRAS*, *TRIM25*, and *PIK3CA*), eight genes have been validated to be associated with AD. *HRAS* was identified as a potential key target of Resveratrol in treatment of AD [39]. In addition, Qu *et al.* found that *HRAS* modified the pathogenic process of AD, indicating that it might serve as a potential therapeutic target for AD [40]. *GRB2* was proved to be a downstream adapter of *ALK* and *RYK*, which exhibit significant functional down-regulation in post-mortem AD [41]. Qian *et al.* reported the correlation between the expression level of immune core genes and the pathological features of AD, and found that *GRB2*, *PIK3R1*, and *KRAS* were immune hub genes associated with Braak stages in AD [42]. The *ELAVL1* expression level was found to be altered in AD and was related to pathological development and cognitive levels [43]. A newly published study reveals that *PTPRG* activates the m^6^A methyltransferase VIRMA to block mitophagy-mediated neuronal death in AD [44]. Meanwhile, a *TRIM25* nonsense mutation (p.C168*) was found to be associated with autosomal dominant early-onset dementia and Parkinsonism, with biomarkers suggestive of AD [45]. Previous research reveals that *PIK3R1* and *PIK3CA* are the most important targets of *BM25*, and *BM25* has been verified to have neuroprotective effects in AD [46]. The above results illustrated the effectiveness of the “prediction” capability of OntoTiger, and the identified candidate AD-related genes have refined the scope for biological experiments and clinical research.

### The Enrichment module characterizes the molecular roles from an ontological perspective

**Figure 4.**
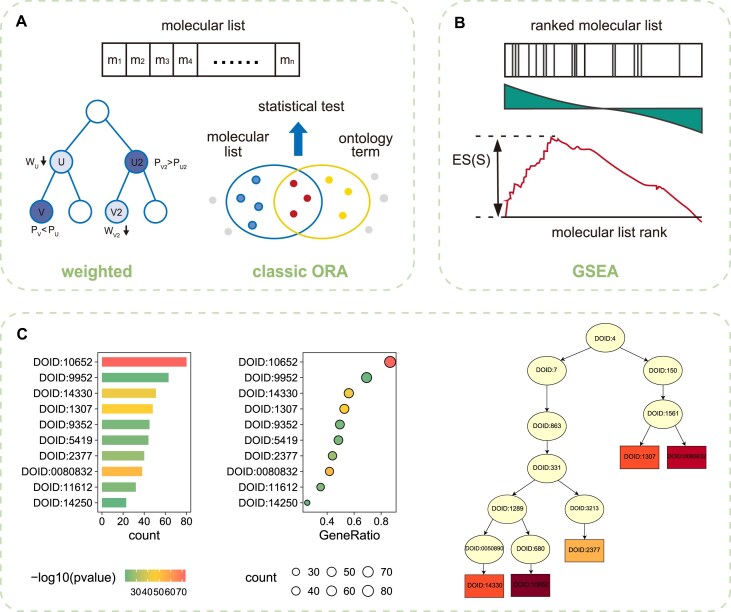
OntoTiger Enrichment analysis module. (**A**) Schematic diagram of the weighted model and the ORA model with an input of a molecular list; (**B**) schematic diagram of the GSEA model with an input of an ordered molecular list; (**C**) the bar plot and point graph of the top 10 enriched terms, and the subgraph of DAG for the top five enriched terms of the case of AD.

OntoTiger provides an Enrichment module to elucidate the potential biological significance of a given molecular list. In this module, three typical enrichment analysis methods are integrated, namely classical ORA, the weighted method, and GSEA (Fig. 4A, B). Generally, different methods will result in different enriched terms due to their different focuses. The Enrichment module supports simple input in the form of a (ordered) molecular list and diverse output in the form of a table and three diagrams (Fig. 4C). The enrichment results mine statistically significant biological process, molecular function, and disease associations from biomedical big data. For example, for a list of DEGs, enrichment analysis can reveal the potential functions of these genes. For patient medication records in electronic health records, enrichment analysis can analyze the association patterns of drugs with diseases or phenotypes, and explore potential treatment strategies or drug repositioning opportunities.

Annotations associate the molecules with ontology terms, allowing for representation of each term as a molecular set. Therefore, the Enrichment module can measure the statistical proportion of an ontology term and a list of molecules, such as DEGs and abundance-varying microbes. For instance, we implemented the DO enrichment analysis by inputting a list of AD-related genes. We collected 91 curated AD-related genes from DisGeNET (Supplementary Data), a comprehensive knowledge platform of disease-associated genes and variants [47]. Both classic and weighted methods were applied with default parameters. The results returned a table of DO terms, ranked by enrichment significance (*P*-value), and three visualized graphs. The top 10 terms of the results are displayed in Table 1. Notably, both methods identified AD as the top DO term, but only five terms overlapped among the top 10 terms. Using the classic method, the top 10 DO terms were concentrated in two branches (Supplementary Fig. S1). Among them, terms such as “Alzheimer's disease”, “tauopathy”, “neurodegenerative disease”, “Parkinson's disease”, and “synucleinopathy” shared an “is_a” relationship, belonging to the branch of “nervous system disease”, along with another term “multiple sclerosis”. On the other hand, terms such as “mild cognitive impairment”, “dementia”, and “cognitive disorder” were grouped in the “disease of mental health” branch. This situation could be caused by the fact that ontology has a hierarchical structure that forms a DAG following the “true-path” rule. The classic method treats each term independently, which can lead to overenrichment of parent terms. In contrast, the weighted method takes the ontology graph topology into consideration. The top 10 DO terms identified with the weighted method, as shown within the DO DAG in Supplementary Fig. S2, were more specific, greatly alleviating the overenriched problem.

**Table 1.** Statistics of the top 10 significant DO terms for AD-related genes with the classic method and weighted method

		Classic			Weighted		
DOID	DO term	Rank	*P-*value	*P*.adjust	Rank	*P*-value	*P*.adjust
DOID:10652	Alzheimer's disease	1	3.33E-71	1.26E-67	1	3.33E-71	1.58E-67
DOID:680	Tauopathy	2	5.28E-71	1.26E-67	–	1	1
DOID:1289	Neurodegenerative disease	3	6.96E-57	1.10E-53	–	1	1
DOID:0080832	Mild cognitive impairment	4	2.18E-52	2.59E-49	2	2.18E-52	5.18E-49
DOID:1307	Dementia	5	2.48E-46	2.36E-43	3	2.48E-46	3.93E-43
DOID:14330	Parkinson's disease	6	3.06E-42	2.43E-39	4	3.06E-42	3.64E-39
DOID:0050890	Synucleinopathy	7	2.86E-41	1.94E-38	–	1	1
DOID:1561	Cognitive disorder	8	1.77E-37	1.05E-34	30	7.95E-17	1.26E-14
DOID:150	Disease of mental health	9	3.12E-33	1.65E-30	260	8.05E-02	6.84E-01
DOID:2377	Multiple sclerosis	10	9.39E-28	4.46E-25	5	9.39E-28	8.92E-25
DOID:5419	Schizophrenia	13	1.88E-25	6.88E-23	6	1.88E-25	1.49E-22
DOID:11612	Polycystic ovary syndrome	15	2.43E-25	7.43E-23	7	2.43E-25	1.65E-22
DOID:9352	Type 2 diabetes mellitus	20	1.78E-22	4.24E-20	8	1.78E-22	1.06E-19
DOID:7148	Rheumatoid arthritis	27	2.90E-21	5.03E-19	9	2.90E-21	1.41E-18
DOID:9952	Acute lymphoblastic leukemia	28	2.96E-21	5.03E-19	10	2.96E-21	1.41E-18

“–” in the Rank column rank represents a non-significant term with a *P*-value of 1.

## Discussion

OntoTiger provides a systematic tool that enables one-stop biomedical ontology-based analysis. It encompasses most common applications of biomedical ontologies, including the qualification of ontology–molecule relationships by annotation, the quantification of the relationships between pairwise ontology terms (or molecules) via similarity calculation, the prediction of molecular roles based on ontology–molecule annotations and ontology similarity network, as well as the elucidation of the potential biological significance for a particular molecular list through enrichment analysis. Compared with other widely used ontology-based tools that focus on specific ontologies and single functions, such as GOSemSim [14], SemFunSim, HPOSim [16], BiNGO [23], GSEA, DOSE [48], TopGO, and DincRNA [21], OntoTiger integrates >20 classic algorithms, supports a variety of molecular types and multiple ontologies, and requires no programming skills. Furthermore, the simple input and diverse data outputs of this platform make OntoTiger more user friendly to bioinformatics community and clinician.

Although OntoTiger provides a valuable biomedical ontology-based tool, further efforts are still needed to extend and enhance its applications. Compared with hundreds of biomedical ontologies, OntoTiger currently only supports GO, DO, and HPO, with a primary focus on *Homo sapiens*. These three ontologies were established early on and boast comprehensive, reliable data and annotations, and they are interoperable with each other. It is worth noting that the OBO Foundry [10] aims at unifying various disparate ontologies, and the Core Ontology for Biology and Biomedicine (COB) [49] aims to provide a common, high-level ontology framework for the biological and biomedical fields. These great efforts led to significant improvements in ontology standardization and applications. We plan to integrate more ontologies from the OBO ontology library and utilize the COB to expand our platform in the future. Another aspect that should be further explored is the integration of more recent methods for functional prediction of molecules and enrichment analysis. In addition, incorporating applications of knowledge graphs and inferring based on biomedical ontologies in further studies will strengthen OntoTiger and make it more comprehensive.

## Supplementary Material

gkaf337_Supplemental_Files

## Data Availability

OntoTiger is freely available at https://bio-computing.hrbmu.edu.cn/OntoTiger. This website is free and open to all users and there is no login requirement.
