# Supplementary material for: OntoTiger: a platform of ontology-based application tools for integrative biomedical exploration
Source: Nucleic Acids Res. 2025 Apr 29;53(W1):W440–50. doi: 10.1093/nar/gkaf337 (PMC12230673; doi:10.1093/nar/gkaf337)
Supplement: gkaf337_Supplemental_Files [file gkaf337_supplemental_files.zip › Supplementary Information.pdf]

Supplementary Materials for

OntoTiger: A platform of ontology-based application tools for integrative biomedical exploration

Haixiu Yang[1,†], Guoyou He[1,†], Meiyi Zhang[1,†], Hongyu Fu[1], Guanzhi He[1], Chao Wang[1], Yangyang Liu[1], Sainan Zhang[1], Tao Wang[2,*], Yongqun Oliver He[3,*], and Liang Cheng[1,4,*]

[1] College of Bioinformatics Science and Technology, Harbin Medical University, Harbin, Heilongjiang, 150081, China.
[2] School of Computer Science, Northwestern Polytechnical University, 1 Dongxiang Rd., 710072, Xi'an, China.
[3] University of Michigan Medical School, Ann Arbor, MI, USA.
[4] National Health Commission (NHC) Key Laboratory of Molecular Probes and Targeted Diagnosis and Therapy, Harbin Medical University, Harbin, 150028, China.

[*] To whom correspondence should be addressed. Tel: +86 153 0361 4540; Email: liangcheng@hrbmu.edu.cn

Correspondence may also be addressed to Tao Wang. Email: twang@nwpu.edu.cn

Correspondence may also be addressed to Yongqun Oliver He. Email: yongqunh@med.umich.edu

[†] The authors wish it to be known that, in their opinion, the first three authors should be regarded as Joint First Authors.

Materials and methods

Similarity calculation of pairwise ontology terms/molecules

Resnik(1), Jiang(2), Lin(3) and Rel(4) are four Information Content (IC)-based methods, which depend on the frequency of the two involved ontology terms and their most informative common ancestor term (MICA) in a specific ontology annotation corpus.

Resnik's method(1)

Resnik's view, the information content (IC) of a term t is defined as following:

$$IC(t) = -\log\frac{n_t}{N} \tag{1}$$

where N denotes the number of different genes related with all the terms, n_t represents the number of different genes related with t. This results in the definition:

$$\text{Sim}_{Resnik}(t_1, t_2) = IC(MICA) \tag{2}$$

Jiang's method(2)

Jiang proposed a method to calculate the semantic distance between terms using the information content (IC):

$$\mathrm{Sim}_{Jiang}(t_1, t_2) = 1 - \min(1, IC(t_1) + IC(t_2) - 2 \cdot IC(MICA)) \quad (3)$$

Lin's method(3)

Lin improved the semantic similarity by normalizing the Resnik semantic similarity using the sum of the information amount of two terms, this approach considers both the same and different information of two terms:

$$Sim_{Lin}(t_1, t_2) = \frac{2 \cdot IC(MICA)}{IC(t_1) + IC(t_2)} \quad (4)$$

Rel method(4)

In order to take relevance information into account, Rel combined Lin's and Resnik's similarity measures, which is defined as follows:

$$Sim_{Rel}(t_1, t_2) = \frac{2 \cdot IC(MICA)(1 - p(MICA)}{IC(t_1) + IC(t_2)} \quad (5)$$

Wang's method(5)

Wang proposed a method to encode an ontology term's semantics (biological meanings) into a numeric value by aggregating the semantic contributions of their ancestor terms (including this specific term) in the ontology graph and, in turn, designed an algorithm to measure the semantic similarity of ontology terms.

Assuming T is the set involving t_1 and all of its ancestor terms of ontology based on 'is_a' relationship. Semantic contribution of term t to t_1 is represented as following:

$$S_{t_1}(t) = \begin{cases} 1 & t = t_1 \\ \max_{t' \in \, children \, of \, (t)} (w \cdot S_{t_1}(t')) & t \neq t_1 \, and \, t \in T \end{cases} \quad (6)$$

where w is semantic contribution factor of semantic association. The value of the summation of all the semantic contributions of T_1 to t_1 is $SV(t_1)$, which is defined as following:

$$SV(t_1) = \sum_{t \in T_1} S_{t_1}(t) \quad (7)$$

Assuming T_2 is the set involving t_2 and all of its ancestor terms, the similarity between t_1 and t_2 is defined as following by Wang's method:

$$Sim_{Wang}(t_1, t_2) = \frac{\sum_{t \in T_1 \cap T_2} \left(S_{t_1}(t) + S_{t_2}(t) \right)}{SV(t_1) + SV(t_2)} \quad (8)$$

SemFunSim method(6)

SemFunSim performs similarity analysis of the functional association of ontology DAGs with term-related genes. Suppose G_1 and G_2 represent the gene sets of t_1 and t_2, respectively. Then SemFunSim describes the similarity between t_1 and t_2 as follows:

$$Sim_{SemFunSim}(t_1, t_2) = SemSim(t_1, t_2) \cdot FunSim(t_1, t_2) \quad (9)$$

where $SemSim(t_1, t_2)$ and $FunSim(t_1, t_2)$ represent semantic similarity based on the ontology DAG, and functional similarity based on term-related genes, respectively. $SemSim(t_1, t_2)$ is described as following:

$$SemSim(t_1, t_2) = \frac{m}{|G_{MICA}|} \cdot \frac{n}{|G_{MICA}|} \tag{10}$$

where $|G_{MICA}|$ represents the number of genes of $MICA$ of t_1 and t_2 , m and n denote the number of genes in G_1 and G_2, respectively. $FunSim(t_1, t_2)$ is described as following:

$$FunSim(t_1, t_2) = \frac{\sum_{i=1}^{m} \max_{1 \le j \le n} \left(Sim(g_{1i}, g_{2j}) \right) + \sum_{j=1}^{n} \max_{1 \le i \le m} \left(Sim(g_{2j}, g_{1i}) \right)}{m + n} \tag{11}$$

Here, $Sim(g_{1i}, g_{2j})$ is the functional similarity between genes g_{1i} and g_{2j}, which is obtained from HumanNet V3.

Onto2Vec method(7)

Onto2Vec is a method for learning dense, vector-based representations of classes in ontologies, as well as biological entities annotated with classes in ontologies, and generating vector representations by combining symbolic reasoning, that is, automated reasoning and statistical representation learning. We first generated a vector-based representation of the classes in the ontology and next obtained the term similarity through the computation of cosine similarity.

OWL2Vec method(8)

The OWL2Vec method is to transform the ontology into a graph, and each term corresponds to the node of the graph. Then the vectorization of the ontology terms is realized by the method of graph embedding, and the term similarity is obtained by calculating the cosine similarity.

Obo2Vec method(9)

The obo2vec method uses a word embedding tool to map GO terms into their semantic vectors. The similarity between terms was then calculated by cosine similarity.

CroGo(10)

CroGO was used to measure the similarity between terms of different GO categories. First, the association between two sets of genes that are annotated to any two given GO terms is calculated. Second, the gene annotations and gene set associations are integrated to calculate the pair-wise term similarity.

To measure the association between two gene sets G_1 and G_2 that are annotated to terms t_1 and t_2 in GO categories C1 and C2 respectively, they define Gene Set Association (GSA) by taking into consideration the weighted edges in a gene co-function network N. GSA is defined as:

$$GSA(G_1, G_2) = \frac{|G_1 \cup G_2| - |G_1 - G_2| - |G_2 - G_1|}{|G_1 \cup G_2|} \tag{12}$$

where G_1 (or G_2) is the set of genes annotated to t_1 (or t_2), $|X|$ represents the size of set X, $G_1 \cup G_2$ is the union of G_1 and G_2, and $|G_1 - G_2|$ is defined as:

$$|G_1 - G_2| = G_1 - \sum_{g_i \in G_1} \left(1 - \prod_{g_j \in G_2} \left(1 - (1 - w_{ij}) \right) \right) \tag{13}$$

where w_{ij} is functional similarity score between genes g_i and g_j in gene co-function network N:

$$w_{ij} = \begin{cases} 1 & i = j \\ 0 & \langle g_i, g_j \rangle \notin N \\ cof(g_i, g_j) & else \end{cases} \tag{14}$$

where $\langle g_i, g_j \rangle$ is an edge in N, and $cof(g_i, g_j)$ is the likelihood of the functional interaction between g_i and g_j in N, and $cof(g_i, g_j) \in [0, 1]$.

Then, given two GO terms t_1 and t_2 from different GO categories $C1$ and $C2$, the term similarity $Sim(t_1, t_2)$ is defined with the integration of GO structure, gene annotations and co-function network:

$$CroGo(t_1, t_2) = GSA(G_1, G_2) \cdot \sqrt{(1 - \frac{|G_1|}{|G_{C1}|}) \cdot (1 - \frac{|G_2|}{|G_{C2}|})} \tag{15}$$

VSM(11)

Vector space models (VSMs) are frequently used in information retrieval for computing the similarity between documents described as vectors of keywords.

A weight is applied to each binary association in order to lower the importance of an association between a GO term and a gene product when a given gene product is associated with many GO terms. This weighting scheme, shown in Eq. (16), is known as inverse document frequency (idf) in information retrieval:

$$idf_j = \log \frac{N}{n_j} \tag{16}$$

Here, the weight of each association between a GO term and gene product j is inversely proportional to the ratio of the number of annotations for this gene product (n_j) to the total number of distinct gene products in the corresponding annotation database (N). Then, each vector is normalized in order to compensate for differences in the number of genes associated with GO terms.

In the VSM paradigm, the similarity between two vectors v_1 and v_2 is usually represented by the angle between these vectors, measured by the dot product of two (normalized) vectors. Any two terms t_1 and t_2 are respectively transformed into vectors v_1 and v_2 according to their annotation information, and their similarity is defined as follows:

$$VSM(t_1, t_2) = \frac{\sum_{i=1}^{n} v_{1i} \cdot v_{2i}}{\sqrt{\sum_{i=1}^{n} (v_{1i})^2} \cdot \sqrt{\sum_{i=1}^{n} (v_{2i})^2}} \tag{17}$$

PBPA and PAPM method(12,13)

The above methods are used for calculating the pair-wise similarity. We utilize the 'pair-wise best pairs-average' (PBPA) method and the 'pair-wise all pairs-maximum' (PAPM) method to calculate the similarity between pair-wise sets based on similarity of pair-wise. Assuming T_1 and T_2 are two sets of terms, with T_1 containing m terms and T_2 containing n terms, the similarity between T_1 and T_2 according to the PBPA method and the PAPM method is defined as Eq. (18) and Eq. (19), respectively.

$$\mathrm{Sim}(T_1, T_2) = \frac{\sum_{i=1}^{m} \max_{1 \le j \le n} \left(Sim(t_{1i}, t_{2j})\right) + \sum_{j=1}^{n} \max_{1 \le i \le m} \left(Sim(t_{2j}, t_{1i})\right)}{m + n} \tag{18}$$

$$\mathrm{Sim}(T_1,T_2)=\frac{\max\limits_{1\le i\le m}\left(\frac{\sum_{j=1}^{n}Sim(t_{1i},t_{2j})}{n}\right)+\max\limits_{1\le j\le n}\left(\sum_{i=1}^{m}\frac{Sim(t_{2j},t_{1i})}{m}\right)}{2} \quad (19)$$

where t_{1i} and t_{2j} represent ith and jth terms of T_1 and T_2, respectively.

Prediction of new molecule-ontology relations

Random walk with restart (RWR) method(14)

Random walk with restart (RWR) method is a global distance metric used to define the similarity between nodes in a network, and it ranks candidate nodes based on this similarity. and to rank candidate nodes on the basis of this similarity to known nodes. In our tool, *OntoTiger*, the RWR method is integrated for predicting molecular-related ontologies and ontology-related molecules. By RWR method the random walker starts on one or several seed nodes and then randomly transits to neighbouring nodes considering the probabilities of the edges between the two nodes. And the probability to return to the seed nodes is supposed as γ. Then, RWR algorithm is defined as following:

$$P_{t+1}=\gamma P_0+(1-\gamma)\mathrm{AP}_t \quad (20)$$

where P_0 denotes the initial probability vector, which changes with the step t and the probability γ, P_t is a vector in which the ith element represents the probability of finding the walker at node i and step t, A is the column-normalized adjacency matrix of the network. The algorithm was performed until the difference between P_t and P_{t+1} falling below 10^{-10}, which means all the nodes become stable.

Enrichment analysis for molecular list

The biomedical ontology enrichment analysis was conducted based on the ontology DAG library, which semantically expands the annotations.

weighted(15)

The weight algorithm of topGO is tailored to detect the locally most significant terms in the GO graph by down-weighting genes in less significant neighbors. We developed this algorithm into universal tool supporting five biomedical ontologies. Based on the ontology DAG with levels, from the bottom to the top, the weighted *p*-value for each node is calculated using a weighted contingency table (Table S1) according to Eq. 21. Then, for each node, compare the significance between the current node and its children (sigRatio is calculated by Eq. 22). If the current node is more significant (sigRatio $<=1$), the weights of relevant molecules annotated with all child nodes are reduced. If any child nodes are more significant (sigRatio >1), the weights of relevant molecules annotated with current node are reduced.

Table S1. Node u and relevant comments in a calculation to identify important molecules

	$sigIterms$	$\overline{sigIterms}$	Sum
$nodeU$	$\lvert sigIterms \cap molecule[U]\rvert$	$\lvert \overline{sigIterms} \cap molecule[U]\rvert$	$molecule[U]$
$\overline{nodeU}$	$\lvert sigIterms \cap \overline{molecule[U]}\rvert$	$\lvert \overline{sigIterms} \cap \overline{molecule[U]}\rvert$	$\overline{molecule[U]}$
Sum	$\lvert sigIterms\rvert$	$\overline{sigIterms}$	$\lvert allmolecules\rvert$

$$\mathrm{X}=\left\lceil \sum_{\mathrm{i}\in\{\mathrm{sigIterms}\cap\mathrm{node(u)}\}}\mathrm{weight[i]}\right\rceil \quad (21)$$

$$\mathrm{sigRatio}=\frac{\mathrm{current.p}}{\mathrm{child.p}} \quad (22)$$

GSEA (16)

The purpose of the Gene set enrichment analysis (GSEA) is to determine whether the annotated molecules of ontology terms are randomly distributed throughout the list of differentially expressed genes or whether they are predominantly concentrated at the top or bottom of the list. We use the R package fgsea to perform GSEA enrichment analysis. The process begins by sorting the molecules-of-interest list based on their scores, followed by an analysis of the distribution of molecules annotated with each ontology term (*P*) within the sorted list (*L*). Finally, the ES score and significance for each ontology term are calculated (Eq. 23 to 25).

The ES score evaluates the weighted sum of molecules in the set S ("hits") and molecules not in S ("misses") at a given position i in the ranked list *L*. P_{hit} represents the weighted sum for molecules in S, while P_{miss} represents the weighted sum for molecules not in S. In the formula $\frac{|r_j|^p}{N_R}$, *p* is the weighting factor. When p=0, the formula reduces to the standard Kolmogorov-Smirnov statistic. When p=1, the denominator of P_{hit} is the sum of all molecule-ontology association strengths in set S, with each molecule-ontology association normalized to this sum. (*r* represents the association strength between a molecule and the ontology of interest, which can be evaluated by FC, etc.)

$$P_{hit}(S,i) = \sum_{\substack{m_j \in S \\ j \le i}} \frac{|r_j|^p}{N_R}, \qquad \text{where } N_R = \sum_{m_j \in S} |r_j|^p \tag{23}$$

$$P_{miss}(S,i) = \sum_{\substack{m_j \notin S \\ j \le i}} \frac{1}{(N - N_H)} \tag{24}$$

$$ES = P_{hit} - P_{miss} \tag{25}$$

Results

Table S2. Annotations of molecules with ontology terms in OntoTiger

Ontology	Molecule	Source of Molecular Description	Method/Source of Annotation	Num. ontology terms	Num. molecules	Num. annotations
MF	pc_gene	GO	-	4,640	18,561	72,330
BP	pc_gene	GO	-	12,261	18,911	138,610
CC	pc_gene	GO	-	1,822	19,925	85,176
DO	pc_gene	GeneRIF	Mgrep/GeneRIF	4,459	15,250	209,776
DO	miRNA	GeneRIF	Mgrep/GeneRIF	844	1,045	16,108
DO	lncRNA	GeneRIF	Mgrep/GeneRIF	477	1,160	5,889
DO	metabolite	HMDB	Mgrep/HMDB	363	22,452	25,886
DO	drug	DrugBank	Mgrep/DrugBank	962	3,130	7,023
DO	microbe	gutMDisorder	Mgrep/gutMDisorder	90	630	1,883
HPO	pc_gene	HPO	-	3,522	14,178	215,840
HPO	miRNA	GeneRIF	Mgrep/GeneRIF	727	1,062	15,418
HPO	lncRNA	GeneRIF	Mgrep/GeneRIF	412	1,122	5,639
HPO	drug	DrugBank	Mgrep/DrugBank	858	1,880	11,416
HPO	microbe	gutMDisorder	Mgrep/gutMDisorder	101	597	2,022

Table S3. Statistics of annotations of "Alzheimer's disease", "tauopathy" and "mild cognitive impairment"

Molecular Type	Alzheimer's disease	tauopathy	mild cognitive impairment
pc_gene	1,388	35	160
miRNA	78	1	6
lncRNA	18	0	1
metabolite	127	0	2
microbe	9	0	0
drug	49	0	2

Table S4. Similarities of AD-tauopathy and AD-MCI pairwise terms

Methods	AD-tauopathy	AD-MCI
Resnik	0.2468777	0
Lin	0.9988084	0
Jiang	0.999411	0.2794333
Rel	0.9061794	0
SemFunSim	0.9922985	0.0003917002
Wang	0.869122	0.1121391
OWL2Vec	0.6866536	0.4874536
Obo2Vec	0.6709766	0.7023126
Ont2Vec	0.9671697	NA

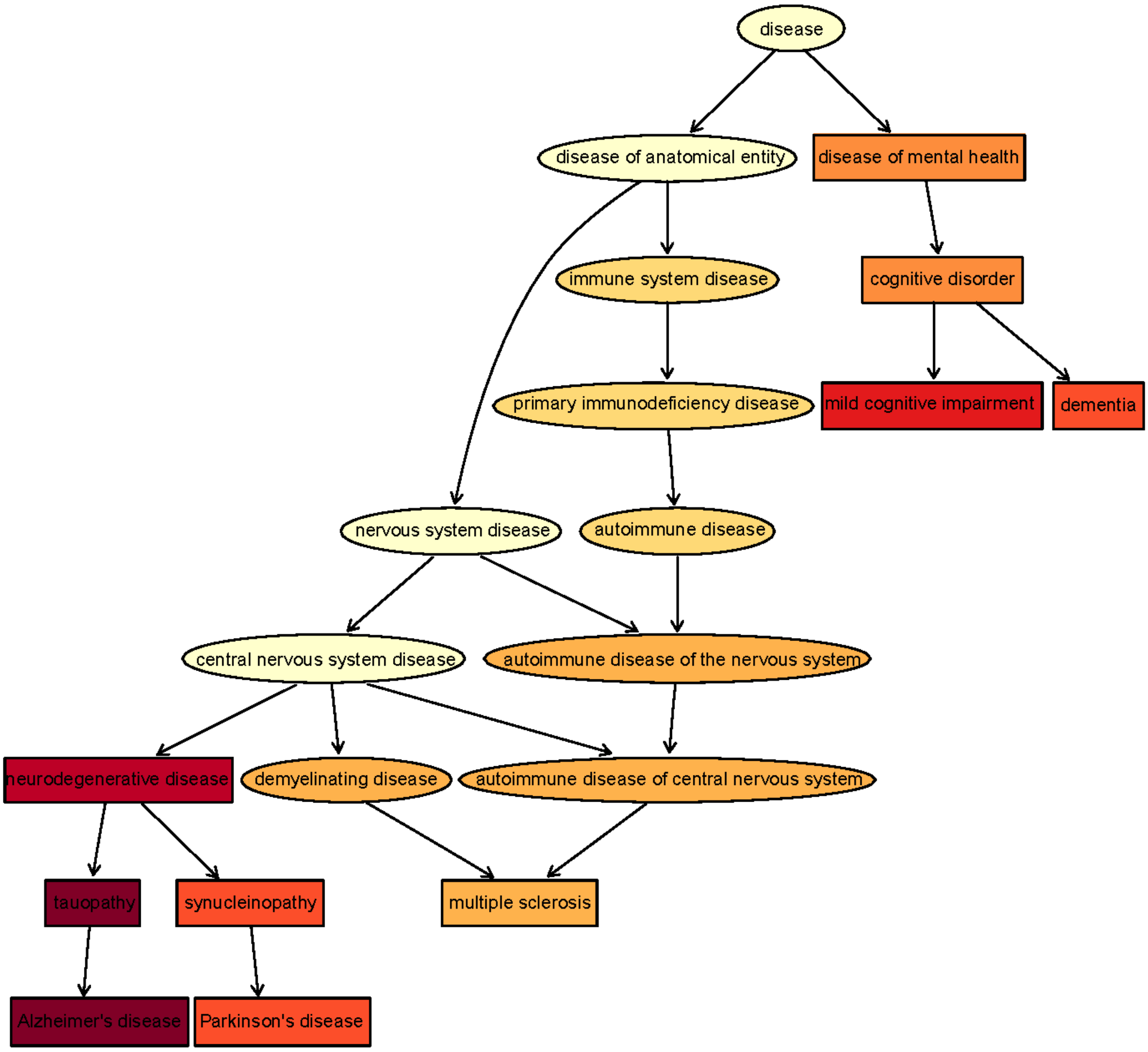

Figure S1. The sub-graph of DAG of top ten enriched terms of the AD case with classical ORA method.

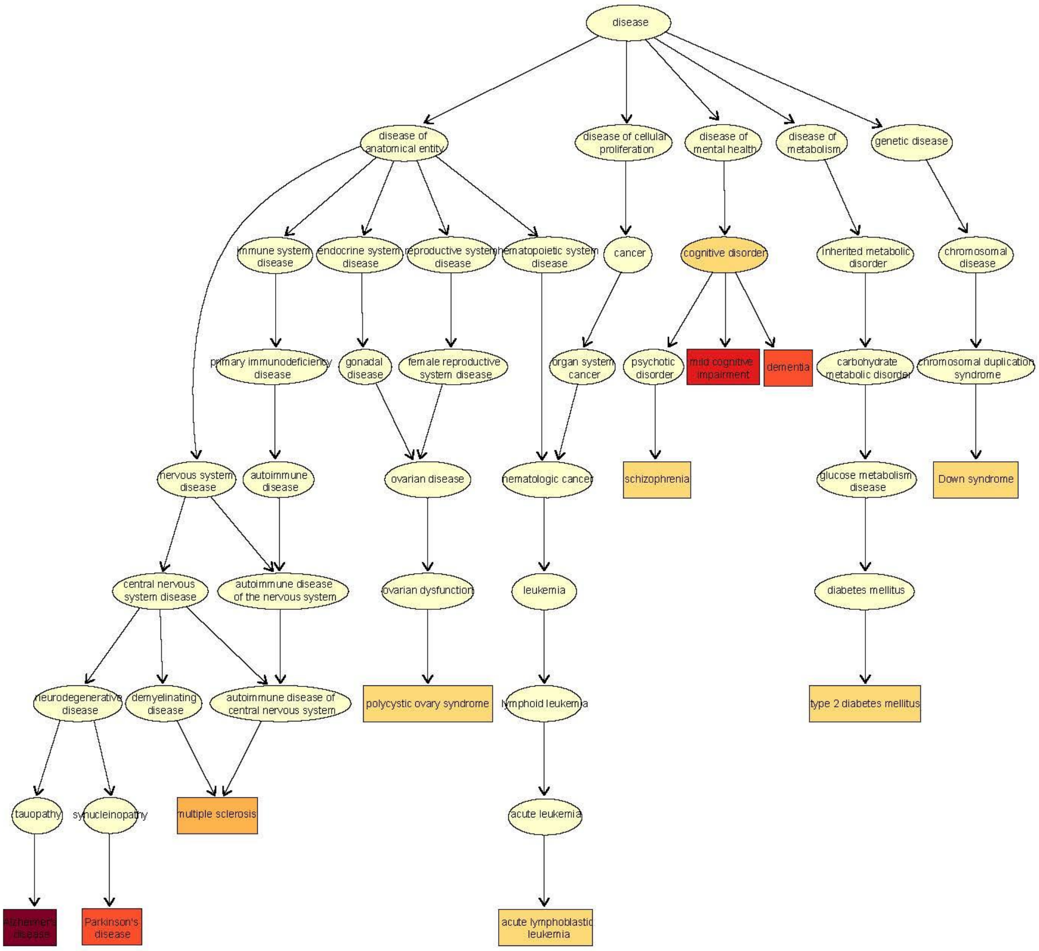

Figure S2. The sub-graph of DAG of top ten enriched terms of the AD case with the weighted method.

References

1. Resnik, P. (1995), *Proceedings of the 14th international joint conference on Artificial intelligence - Volume 1*. Morgan Kaufmann Publishers Inc., Montreal, Quebec, Canada, pp. 448–453.
2. Jiang, J.J. and Conrath, D.W. (1997), *Proceedings of the International Conference on Research in Computational Linguistics (ROCLING)*. Taiwan.
3. Lin, D. (1998), *Proceedings of the Fifteenth International Conference on Machine Learning*. Morgan Kaufmann Publishers Inc., pp. 296–304.
4. Schlicker, A., Domingues, F.S., Rahnenfuhrer, J. and Lengauer, T. (2006) A new measure for functional similarity of gene products based on Gene Ontology. *BMC Bioinformatics*, **7**, 302.
5. Wang, J.Z., Du, Z., Payattakool, R., Yu, P.S. and Chen, C.F. (2007) A new method to measure the semantic similarity of GO terms. *Bioinformatics*, **23**, 1274-1281.
6. Cheng, L., Li, J., Ju, P., Peng, J. and Wang, Y. (2014) SemFunSim: a new method for measuring disease similarity by integrating semantic and gene functional association. *PLoS One*, **9**, e99415.
7. Smaili, F.Z., Gao, X. and Hoehndorf, R. (2018) Onto2Vec: joint vector-based representation of biological entities and their ontology-based annotations. *Bioinformatics*, **34**, i52-i60.

8. Zhao, L., Sun, H., Cao, X., Wen, N., Wang, J. and Wang, C. (2022) Learning representations for gene ontology terms by jointly encoding graph structure and textual node descriptors. *Brief Bioinform*, **23**.
9. Zhao, L., Wang, J., Hu, Y. and Cheng, L. (2020) Conjoint Feature Representation of GO and Protein Sequence for PPI Prediction Based on an Inception RNN Attention Network. *Mol Ther Nucleic Acids*, **22**, 198-208.
10. Peng, J., Chen, J. and Wang, Y. (2013) Identifying cross-category relations in gene ontology and constructing genome-specific term association networks. *BMC Bioinformatics*, **14 Suppl 2**, S15.
11. Bodenreider, O., Aubry, M. and Burgun, A. (2005) Non-lexical approaches to identifying associative relations in the gene ontology. *Pac Symp Biocomput*, 91-102.
12. Wang, D., Wang, J., Lu, M., Song, F. and Cui, Q. (2010) Inferring the human microRNA functional similarity and functional network based on microRNA-associated diseases. *Bioinformatics*, **26**, 1644-1650.
13. Pesquita, C., Faria, D., Falcao, A.O., Lord, P. and Couto, F.M. (2009) Semantic similarity in biomedical ontologies. *PLoS Comput Biol*, **5**, e1000443.
14. Kohler, S., Bauer, S., Horn, D. and Robinson, P.N. (2008) Walking the interactome for prioritization of candidate disease genes. *Am J Hum Genet*, **82**, 949-958.
15. Alexa, A., Rahnenführer, J. and Lengauer, T. (2006) Improved scoring of functional groups from gene expression data by decorrelating GO graph structure. *Bioinformatics*, **22**, 1600-1607.
16. Subramanian, A., Tamayo, P., Mootha, V.K., Mukherjee, S., Ebert, B.L., Gillette, M.A., Paulovich, A., Pomeroy, S.L., Golub, T.R., Lander, E.S. *et al.* (2005) Gene set enrichment analysis: a knowledge-based approach for interpreting genome-wide expression profiles. *Proc Natl Acad Sci U S A*, **102**, 15545-15550.
